# Evaluation of Robotic Swabbing and Fluorescent Sensing to Monitor the Hygiene of Food Contact Surfaces

**DOI:** 10.3390/foods14193311

**Published:** 2025-09-24

**Authors:** Siavash Mahmoudi, Clark Griscom, Pouya Sohrabipour, Yang Tian, Chaitanya Pallerla, Philip Crandall, Dongyi Wang

**Affiliations:** 1 Department of Biological and Agricultural Engineering, University of Arkansas, Fayetteville, AR 72701, USA; siavashm@uark.edu (S.M.); yangtian@uark.edu (Y.T.); 2 Department of Food Science, University of Arkansas, Fayetteville, AR 72701, USA; crgrisco@uark.edu (C.G.);

**Keywords:** environmental monitoring, environmental swabbing, robotics in food safety, precision of human versus robot

## Abstract

Effective environmental monitoring is critical for preventing microbial and allergenic cross-contamination. However, manual swabbing methods, commonly used to verify hygienic conditions, are prone to inconsistent results because of variability in pressure, coverage, and techniques. Two novel solutions will be explored to address these challenges: a robotic swabbing system with tactile sensing control, and a fluorescence/absorbance spectrometer for non-contact, protein-based residue detection. The robotic system was evaluated against trained and untrained humans, measuring water pickup, surface coverage, and pressure consistency. Concurrently, the fluorescence system analyzed model poultry protein soil to correlate spectral patterns with contamination levels. The robotic system demonstrated statistically superior performance, achieving consistent force application and near-complete surface coverage, overcoming key limitations of manual sampling. The fluorescence system distinguished contamination with high sensitivity, validating its use as a rapid, non-contact assessment tool. Together, the robotic sample acquisition and the spectrometer’s sensitive analysis provide a dual-modality framework for enhancing hygiene monitoring in manufacturing facilities.

## 1. Introduction

The Centers for Disease Control and Prevention (CDC) estimates that food contaminated by microbial pathogens has caused an estimated 9.9 million human illnesses, from which more than 53,000 people have been hospitalized, and nearly 1000 deaths have occurred; more specifically, 931 deaths have occurred [1]. Unfortunately, outbreak data summarized by Nazir et al. [2] showed that foodborne illness from eating contaminated foods is on the rise. To reverse these trends, food processors must redouble their efforts to reduce microbial contamination, much of which comes from exposure to dirty food contact surfaces [3]. Food-processing facilities follow their written Sanitation Standard Operating Procedures (SSOP) that includes routine environmental monitoring procedures (EMPs) to identify potential sources of microbial contamination and validate the efficacy of prior cleaning and sanitation [4]. According to the Food Safety Modernization Act [5], no sampling tool is specified for environmental monitoring regulations, but swabs and sponges are recommended [6].

Adherence to U.S. Food and Drug Administration (FDA)/ U.S. Department of Agriculture (USDA) regulations and standards is not merely a compliance exercise; it is absolutely essential for minimizing the risks of foodborne illness outbreaks, preventing cross-contamination, enhancing product traceability, and safeguarding consumers’ health while maintaining producers’ brand integrity. These routine risk-reduction programs depend on surface swabbing to accurately assess contamination risks, validate the effectiveness of their SSOP protocols, and ensure the overall safety of the production environment. Effective swabbing is a primary source of data that provides foundational information upon which these controls are built and routinely validated. The high incidence of foodborne illnesses and the documented persistence of pathogenic biofilms have established undeniable and continuous threats. Regulatory bodies have responded by mandating preventative environmental monitoring as a key control measure.

Consequently, the quality and reliability of the swabbing processes dictate the efficacy of the entire proactive safety protocols. This elevates environmental swabbing from a mere procedural task to a cornerstone of public health and industrial safety, where its accuracy directly influences the ability to preemptively identify and mitigate contamination risks through measures like trend analysis and sanitation validation. Despite their widespread and long-standing application, traditional manual swabbing techniques are intrinsically hampered by several critical limitations, including significant human operator-dependent variability, often suboptimal microbial recovery efficiency, and the poor reproducibility of the results [7]. Such inherent constraints markedly reduce the confidence that should be placed in these contamination monitoring outcomes [6]. More critically, they elevate the risk of underreporting or mischaracterizing microbial loads on food contact surfaces, potentially leading to false assurances of safety.

The immense variability across surface sampling is due to variability among technicians, low recovery rates, and minimal reproducibility [8,9]. A substantial body of research has consistently demonstrated inconsistencies in fundamental swabbing parameters such as the pressure applied by the operator, the angle of the swab relative to the surface, the specific swabbing pattern employed, and overall technique all significantly contribute to errors in sample collection [10,11].

USDA/Food Safety and Inspection Service (FSIS) requires that food contact surfaces and equipment in Zone 1 be “clean to sight and touch”, free of visible dirt or residual food, and feel clean and be free of food residues when touched [12]. Environmental monitoring is usually performed by using swabs or sponges, but this can result in low microbial recoveries, below 2.5 CFU/cm^2^ [13]. Despite their widespread use, swabs and sponges have notable limitations. Swabs are less effective for large or rough surfaces, while sampling contact-type plates are limited to sampling flat areas. Recovery rates can vary depending on surface type, moisture, and the presence of chemical sanitizers, potentially affecting the accuracy of the results [14]. Therefore, there is a clear need for the development and broader adoption of better tools beyond traditional swabs and sponges to enhance environmental monitoring, particularly for applications requiring higher sensitivity, rapid responses, or coverage of complex surfaces.

From another perspective, many foods supply proteins that contain essential amino acids that humans cannot synthesize in their bodies but must be supplied by the foods we eat. Chickens and eggs provide high-quality proteins that contain the essential amino acids such as tryptophan, tyrosine, and phenylalanine [15]. The concentrations of these free amino acids in the skeletal muscles of 6-week-old chicks were 0.04 nmol/mg tissue of tryptophan, 0.14 nmol/mg tissue tyrosine, and 0.19 nmol/mg tissue phenylalanine [16]. These amino acids show natural fluorescence, with the fluorescence of tryptophan being the most prominent [17].

Beyond performance variability, manual swabbing carries recurring labor and training costs that scale with sampling frequency. Automating swab application can shift a portion of this workload from manual execution to supervised operation. As an order-of-magnitude reference in the U.S. (2025), a collaborative arm with a simple with sensors and basic mounting is on the order of USD 40–50k upfront, based on current vendor list pricing [18]. Routine maintenance is light and centered on periodic visual/functional checks with a recommended bi-annual service inspection; approved cleaning agents include water, isopropyl alcohol, and dilute ethanol (direct bleach on the bare arm is discouraged) [19]. Because the arm is IP54, it should be located outside direct wash-down or fitted with a protective cover when harsher sanitation is required [20,21]. The control stack described here is robot-agnostic and can be ported to washdown-rated manipulators (IP66–IP69K) commonly used in food plants with minimal changes to kinematic interfaces and I/O wrappers; calibration is repeated once per tool configuration. Operationally, deployment follows standard collaborative-robot practice: perform a risk assessment per ISO 10218 and ISO/TS 15066, complete mounting, connect basic I/O or a PLC, and program via the teach pendant [22,23]. No prior robotics background is required for basic operation; operators and technicians can be trained using freely available UR Academy e-learning and a short core course [24].

By comparison, the fully loaded annual cost of one environmental monitoring technician is approximately USD 85–90k when typical base wages (∼$59–63 k/year) are adjusted using the Bureau of Labor Statistics Employer Costs for Employee Compensation (wages ≈70%, benefits ≈30%) [25,26,27]. Thus, the capital outlay for a single robotic swabbing cell is broadly comparable to or less than one technician-year, noting that site-specific integration and safety provisions determine the final total.

The purpose of this preliminary research was to evaluate the feasibility of a more precise robot swabbing method, together with a non-contact fluorescence spectrometer, to detect food soil on typical food contact surfaces for in-plant evaluations and reproducible environmental sampling systems. Combining a rapid soil detection system with a rapid and precise robotic swabbing system could result in a significant improvement in routine cleaning and sanitation as well as producing a plant-wide sanitation validation map for production managers and third-party auditors.

## 2. Materials and Methods

### 2.1. Robotic Swabbing

#### System Overview

**Figure 1:**
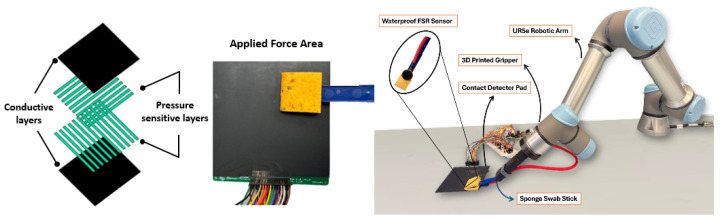
(**Left**) A schematic of the layered structure consisting of pressure-sensitive and conductive layers forming the sensing matrix. The Universal Robot 5e robotic arm (**Right**) is equipped with a 3D printed holder holding a 3M sponge swab stick embedded with a waterproof force-sensing resistor (FSR). The swabbing motion is applied over a contact detection pad (10 cm × 10 cm tactile sensor array), enabling real-time feedback on pressure distribution and surface coverage [28].

This robotic system integrated a Universal Robots UR5e robotic arm https://www.universal-robots.com/products/ur5e/ (accessed on 23 September 2025) (Universal Robots A/S, Odense, Denmark) with a custom-designed gripper for holding a deformable sponge swab, commonly used in food safety applications (3M, Maplewood, MN, USA). Embedded within the sponge were single-cell waterproof force-sensing resistors (FSR; Sensitronics, Skagit County, WA, USA) that provided a real-time feedback control loop to maintain contact pressure during swabbing. To assess macro-scale coverage, a 10 × 10 cm tactile pad (Shunt Mode Matrix Array, Sensitronics, Skagit County, WA, USA) incorporating a 16 × 16 FSR grid recorded the contact location and pressure distribution across the swabbing trajectory, as shown in Figure 1.

This dual-sensor setup enabled both micro-level force tracking at the point of the sponge’s contact of the surface and macro-level surface coverage analysis based on previous research [28]. Each trial began with precisely 1.00 mL of water being applied to the contact detector pad, and the robot executing a predefined zigzag swabbing pattern across the target area. The tactile feedback was essential not only for regulating applied force but also for detecting any missed spots, ensuring complete surface sampling, a limitation frequently observed in human swabbing [29].

To address the challenges associated with Deformable Tool Manipulation (DTM), the system employed a State-Adaptive Koopman Linear Quadratic Regulator (SA-KLQR) control framework [28]. This control approach was designed to handle the nonlinear and time-varying dynamics introduced by the deformable nature of the swab and its interaction with variable surface conditions. The SA-KLQR controller linearized the system dynamics using the Koopman operator, enabling optimal control through a Linear Quadratic Regulation (LQR).

The Mentioned controller follows a dual-loop architecture: an inner loop regulates normal contact force to a target band, while an outer loop tracks the zigzag trajectory for uniform area coverage. For deformable tools manipulation, the interaction dynamics are nonlinear and state-dependent [30]. The robot’s contact-rich dynamics are lifted to a linear representation in an observable space (Koopman framework), enabling optimal state-feedback on the lifted system, with real-time switching among local linear models along the path [31]. A full derivation and ablation are reported in our prior robotics study [28]. The brief introduction of the control algorithm are introduced below.

The swab is a soft, wetted tool whose stiffness and contact geometry change with angle, speed, and saturation. This creates interaction dynamics that are nonlinear and that vary along the path. Rather than build a single complex nonlinear model, we learn a set of simple, data-driven linear surrogates in a higher-dimensional “observable” space. In practice, we transform the measured states and inputs into a compact set of features (polynomials plus a few radial-basis functions) and use Extended Dynamic Mode Decomposition to identify how these features evolve from one control step to the next [32]. The result is a small bank of local linear models that are accurate within their region of operation.

On top of each local model we apply standard linear–quadratic regulation. The feedback law penalizes force error and unnecessary motion, so the controller tracks the coached contact band while avoiding aggressive inputs. An integral term removes steady bias caused by sponge compliance or slow drift. This choice gives predictable behavior with a small number of tuning weights and makes the procedure easy to reproduce across labs [33]. Because contact properties change as the zigzag progresses, the controller selects the nearest local model online and blends during handover to keep commands smooth. This state-adaptive scheme reduces tool pivoting and maintains uniform contact over the full area.

Two design points make the approach practical. First, all models are identified directly from short calibration logs, which avoids bespoke mechanics models of the swab. Second, only three items need refreshing when the swab material, size, or wetness changes: the short data collection, the local model fit, and the thresholds used by the centroid-based stabilizer. The core tuning rules remain the same. Full derivations and ablation studies for the state-adaptive Koopman LQR controller are provided in our prior technical paper, which this work builds upon [28]. Section 2.2 explains how the coached force band is obtained from the ADC–force calibration.

The robot dynamically adjusted its end-effector pose and movement to maintain the desired force based on the output of the Analog-to-Digital Converter (ADC), a value determined through preliminary calibration experiments. ADC values are expressed in unitless digital values rather than conventional physical units like volts. In these trials, the contact force was incrementally increased while measuring both liquid recovery and swab deformation. A centroid-based fuzzy force regulation algorithm further refined the control by centering the contact force application and minimizing tool pivoting and distortion. This coordination of tactile sensing, optimal control, and fuzzy regulation leads to uniform swabbing force and enhanced recovery.

### 2.2. Force–ADC Correlation and Mapping

The unitless ADC readouts from the embedded swab force sensor and from the 16 × 16 tactile pad were mapped to physical force (N) using a bench calibration with a TMS–Pro Advanced Texture Analyzer (Food Technology Corporation, VA, USA) equipped with a NIST-traceable load cell. The analyzer applied a quasi-static ramp from 0 to 20 N over 200 s with a flat plate, while ADC streams from both sensors were logged synchronously.

To account for spatial non-uniformity, per-cell calibration was performed by loading a geometry-controlled footprint and recording the individual cell responses across the 10 cm × 10 cm array. A global mapping was then obtained by taking the median fit across cells and retaining cell-specific correction factors used during post-processing of coverage and pressure maps.

The same 0–20 N ramp was repeated with the sponge swab mounted in the experimental configuration and wetted to the same state used during trials, thereby capturing the compliance and hydration effects of the deformable interface. This calibration produced the reference used to coach participants to maintain the target range of 270–310 ADC.

For both sensors, we fitted a monotone, invertible relation through Equation (1):

(1) F=M(ADC),

implemented as a shape-constrained (isotonic/piecewise-linear) fit, which accommodates the mild nonlinearity and hysteresis expected in soft contacts. As anticipated, the calibration revealed a low-load under-registration region (preload/creep) and a high-load over-registration region (growth of effective contact area through the wetted sponge). To ensure repeatability in practice, the coaching window of 270–310 ADC was selected from the mid-range of the fitted curve, where the deviation band is minimal and the mapping is most stable to small perturbations.

The tactile pad’s 0.2 N criterion refers to per-cell activation. During swabbing, multiple adjacent cells typically exceed this threshold simultaneously; hence, aggregate surface contact during robot and human trials is substantially above 0.2 N while the swab FSR is maintained within the calibrated 270–310 ADC window.

**Figure 2:**
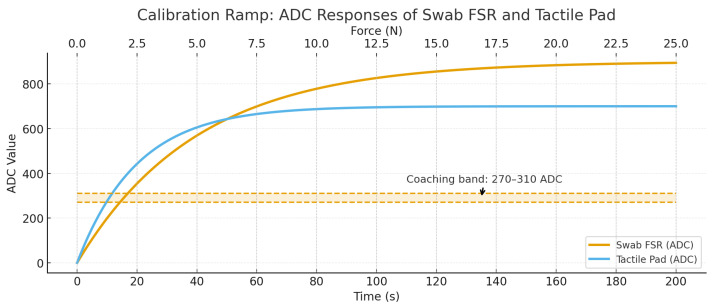
Calibration ramp showing ADC responses from the embedded swab FSR and the tactile pad during a 0–25 N, 200 s force ramp. The shaded band with dashed bounds indicates the coaching window (270–310 ADC) used to guide participants during trials.

Before each calibration sequence, the load cell was zeroed and the sensors were equilibrated under no load. Manufacturer-specified load-cell accuracy and ADC quantization were propagated through M(·) to generate the shaded deviation band reported with the calibration curve. Repeated ramps yielded consistent mappings (visual agreement within the deviation band), supporting the robustness of the selected operating window. The full calibration curve and bench setup are shown in Figure 2.

The tactile pad and the embedded swab FSR exhibit distinct ADC–force trajectories because of their sensor structures and the viscoelastic dynamics of the wetted sponge. The tactile pad is a laminated, shunt-mode matrix in which many microcontacts form in parallel; small normal loads rapidly bridge resistive layers across multiple cells, yielding a comparatively steep low-load response but an early approach to saturation as the microstructures collapse [34]. In contrast, the swab FSR is a single-element film loaded through a compliant, fluid-saturated sponge. The sponge introduces damping and creep (Kelvin–Voigt–like behavior) [35], spreading force over time and reducing the effective pressure at the FSR at low loads under-registration [36]. As load increases, the true contact area and liquid redistribution within the sponge grow, transferring a larger fraction of the normal force to the FSR; this produces the slower initial rise followed by a higher asymptote observed for the swab trace [37]. The combination of array saturation in the pad and viscoelastic mediation in the sponge explains the earlier plateau of the pad curve and the delayed but ultimately greater ADC values of the swab FSR.

#### 2.2.1. Swabbing Force Regulation and System Calibration

To limit tool pivoting and maintain uniform contact During swabbing, the 16 × 16 tactile pad provides a pressure/force map $f_{ij}$ over the 10cm×10cm area. SA-KLQR controller computes the force-distribution centroid $\left(C_{x},C_{y}\right)$ and its deviation *D* from the desired pad center $\left(C_{x}^{*},C_{y}^{*}\right)$, using Equation (2) as below:

(2) $$C_{x}=\frac{\sum_{i}\sum_{j}x_{i}f_{ij}}{\sum_{i}\sum_{j}f_{ij}},\;C_{y}=\frac{\sum_{i}\sum_{j}y_{j}f_{ij}}{\sum_{i}\sum_{j}f_{ij}},\;D=\sqrt{{\left(C_{x}-C_{x}^{*}\right)}^{2}+{\left(C_{y}-C_{y}^{*}\right)}^{2}}.$$

In parallel, controller monitors the temporal regularity of the centroid-error signal D(t) using a short sliding-window *Fuzzy Entropy* metric [38], denoted FuzzyEn(D). When either (i) the centroid deviation exceeds a data-driven threshold $D>\tau_{D}$, or (ii) the entropy indicates unstable contact $\mathrm{FuzzyEn}\left(D\right)>\tau_{E}$, the controller applies a small quaternion-based roll-angle correction Δϕ and modulates the force command ΔF to re-center contact and suppress pivot-induced deformation. These corrections are bounded by the SA–KLQR weights to preserve the nominal trajectory and force setpoint.

For each swab type (material, size) and wetness condition, we perform a brief single-pass calibration at the target nominal force. In this pass, the robot executes the standard zigzag over the tactile pad while $f_{ij}\left(t\right)$ and D(t) are logged. From the resulting centroid trajectory, we estimate statistics under stable contact (median and 95th percentile of *D*) and set the centroid-deviation threshold $\tau_{D}$ to the upper bound of this stable range to avoid triggering on quantization or sensor noise. Over the same window, we compute the fuzzy-entropy metric FuzzyEn(D) of the centroid-error signal and choose the entropy threshold $\tau_{E}$ to detect the onset of irregular contact dynamics. Finally, we refresh the embedded FSR force–ADC mapping at the chosen nominal force to compensate for sponge/handle compliance and fluid uptake. This calibration yields task-specific thresholds without retuning the optimal controller.

At runtime, the centroid regulator runs at the same servo rate as the force loop. When a trigger condition is detected, a bounded corrective action (Δϕ,ΔF) is injected and exponentially decayed back to zero once $D\leq \tau_{D}$ and $\mathrm{FuzzyEn}\left(D\right)\leq \tau_{E}$, preventing limit cycles while maintaining uniform contact.

#### 2.2.2. Controller Hyperparameters and Reproducibility

The controller parameters fall into five coherent groups: (i) timing and data acquisition, (ii) Koopman lifting and identification, (iii) local-model bank and switching, (iv) optimal regulation weights, and (v) centroid-based contact stabilization. We report not only the settings but also the rule by which they were selected so that the procedure transfers across swab media and surface types.

In timing and data acquisition, the servo-loop frequency $f_{\mathrm{servo}}$ was chosen to exceed the dominant contact bandwidth yet remain within the UR5e real-time budget. Tactile-pad and embedded FSR streams were sampled synchronously and denoised with a short moving window that removes ADC quantization without introducing measurable phase lag. The effective acquisition rates and filter lengths are reported in Table 1.

Koopman lifting and identification were configured to yield a compact yet predictive surrogate. We used a small observable dictionary Ψ(·), low-order polynomials augmented with a limited set of radial-basis functions distributed along the planned path, and identified the lifted linear model via EDMD with ridge regularization to suppress overfitting. The lifted dimension dim(Ψ) and the EDMD window length were selected by minimizing one-step prediction error on calibration ramps (Section 2.2), with preference given to the smallest model that preserved stable prediction.

Because contact stiffness and pose coupling vary along the zigzag, we employed a local-model bank with smooth switching. The path was partitioned into $N_{\mathrm{reg}}$ regions, each with its own local linear model. At runtime, the controller selects the nearest region in state space with a small hysteresis buffer, and blends commands with factor β to avoid discontinuities during transitions. This scheme retains the simplicity of linear feedback while adapting to state-dependent interaction.

Optimal regulation weights were set by validation on scripted contact ramps and uniform swabbing traces. The LQR matrices (Q,R) balance force regulation against pose drift and input effort; we performed a small grid search and selected the pair that minimized force RMSE while avoiding limit cycles. An integral term on force error ($K_{I}$) was increased until steady-state error was removed without overshoot.

Finally, centroid-based contact stabilization is governed by two thresholds that are derived from a brief, task-specific calibration. The centroid deviation limit $\tau_{D}$ is set to the 95th percentile of D(t) under stable contact, and the entropy threshold $\tau_{E}$ marks the onset of irregular FuzzyEn(D) dynamics in the same window. When either trigger fires, small, bounded corrections (Δϕ,ΔF) are injected into the SA–KLQR loop and then exponentially decayed once contact re-centers.

All runtime settings used in this study are summarized in Table 1; when the swab material, geometry, or wetness changes, the EDMD fit, the small model bank, and the data-driven thresholds $\left(\tau_{D},\tau_{E}\right)$ are refreshed, while the selection procedure remains unchanged [28].

### 2.3. Fluorescence Spectroscopy for Detecting Protein-Based Contamination

#### 2.3.1. Sample Preparation

Preliminary experiments had shown that a model food soil suspension of raw chicken thigh meat, fat and skin possessed natural fluorescence. Raw bone in chicken thighs were purchased from the local grocery store. Dilutions were created by blending chicken and tap water with a Ninja 72 oz countertop blender (Ninja Professional Blender 1000W 72-oz. BL610, Needham, MA, USA) To determine the limit of detection (LOD), the raw chicken and tap water were tested at multiple dilution ratios (1:10 to 1:100). Gravity separation in a refrigerator at 38 °F was used to settle out the fat particles leaving a more homogeneous solution. After settling, the middle layers of the mixtures were pipetted into 3.5 mL cuvettes and tested using the fluorescence instrument.

#### 2.3.2. Spectral Analysis

The Horiba, Duetta florescence and absorbance spectrometer (https://www.horiba.com/usa/scientific/products/detail/action/show/Product/duetta-1621/ (accessed on 23 September 2025)) was used to generate excitation and emission fluorescence spectra collected over the range of 240–305 nm, with the strongest emission levels being observed at and excitation 280–290 nm, likely from the naturally occurring tryptophan amino acid residues [39]. The specifications for measuring the excitation were an excitation range of 240–305 nm, a band pass of 10nm, an integration time of 0.05 s, and 5 detector accumulations. Absorbance measurement specifications included a scan from 230–550 nm with 5 nm step increments, a 0.01 s integration time, and a 2 nm band pass. For the absorbance measurements, a blank of tap water was used before sample measurements were taken; software supplied with the instrument was used to record and analyze the absorbance and fluorescence spectra.

The fluorescence and absorbance spectrometer specifications include a fluorescence intensity of water Raman SNR > 6000: 1 RMS, 350 nm excitation, and 5 nm silts. A CDD/spectrograph and 75 W Xenon arc lamp was used. For the EzSpec software, a Windows 10 or 11 64 bit OS laptop or PC with 1 USB port is required. Fluorescence and absorbance values of tap water and chicken soil dilutions (1:25, 1:50, and 1:100) were compared using the spectrometer. All statistical analysis was completed in R [40]. Group comparisons were tested using pairwise Tukey’s Honest Significant Difference (HSD) tests with a significance level of *p* < 0.05.

The selection of dilution was based on using our senses of following USDA guidelines of clean to sight and touch. The limit of detection for human sensory perception in a laboratory setting was a 1:50 dilution. Chicken-processing plants are dark, wet, that can potentially limit human sensory abilities, causing an even higher limit of detection [41].

### 2.4. Segregation and Cross-Contamination Controls

To manage cross-contamination between the non-contact fluorescence measurements and the robot-assisted contact swabbing, we separated workflows and used single-use consumables with validated sanitation. Fluorescence/absorbance readings were taken on a bench-top spectrometer using sealed cuvettes; no poultry dilutions or open vessels were present in the robot area [42]. Swabbing media were single-use sterile sponges mounted in a cleanable or autoclave-compatible holder, and the holder plus any contact surfaces were sanitized between sites with facility-approved agents. To integrate fluorescence with robotic swabbing, the collected sample was expressed from the sponge into a sterile bag, transferred to a sterile tube, and then pipetted into a UV-transparent cuvette for measurement. These controls align with ISO surface-sampling practice and regulatory expectations for CGMP sanitation and preventive controls [42,43,44,45].

### 2.5. Experimental Design: Robotic and Human Swabbing

This experiment compared human versus robotic swabbing efficiency in a side-by-side environmental sampling task, evaluating three performance metrics—water pickup efficiency, surface coverage consistency, and pressure uniformity—to test the hypothesis that the robot’s contact force-feedback control loop would yield more reliable results than either the untrained or trained human operators.

**A.**  **Human Swabbing Procedure**

Ten human volunteers each performed six swabbing trials: three conducted as untrained operators, followed by three additional trials after standardized training. The study protocol was reviewed and approved by the University Human Subjects Institutional Review Board (Approval No. 2406545996).

For the untrained trials, exactly 1 mL of water was pipetted onto a 10 × 10 cm tactile pad using a calibrated volumetric pipette (Socorex Acura E-25, Ecublens, Switzerland). Each 3M sponge was weighed in its dry state on an analytical balance (Sartorius Balance, Göttingen, Germany) to four decimal places, and then reweighed following completion of each swabbing trial. Participants were instructed simply to “swab the surface with this 3M sponge in any zigzag pattern you choose.” Each volunteer completed three consecutive swabs under these untrained conditions.

Following the initial trials, participants underwent a 15-min standardized training protocol. Training began with a visual demonstration in the form of a video showing the ideal zigzag swabbing pattern, including complete coverage of the tactile pad with approximately 5 mm lateral overlap between passes [46]. This was followed by guided hands-on practice using a dry tactile pad, during which the experimenter provided real-time feedback on maintaining the correct angle (45° relative to the surface), swabbing speed (2 cm/s), and applied pressure, the latter demonstrated using a hand-held force gauge. To further reinforce pressure control, participants observed a live display of ADC force sensor readings and were coached to maintain the sponge force within the target range of 270–310 ADC.

After completing training, each participant performed three additional swabbing trials under identical conditions to the untrained sessions, with the exception that they were instructed to apply the trained zigzag pattern while adhering to the specified angle, speed, and target pressure range.

**B.**  **Robotic Swabbing Procedure**

**Figure 3:**
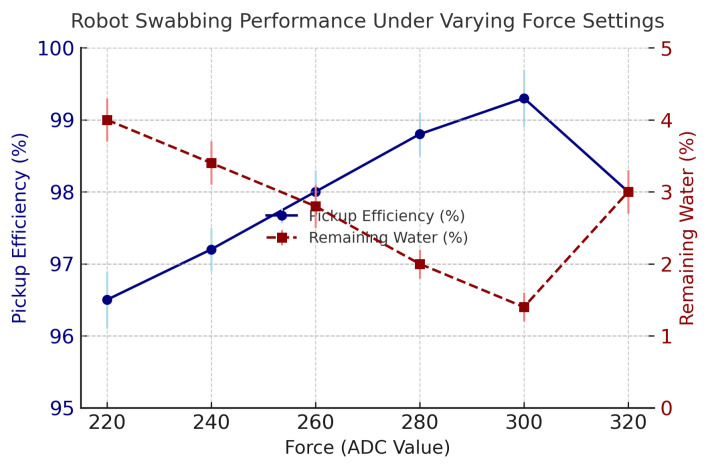
Robot swabbing performance under varying force settings. The plot shows pickup efficiency (blue solid line) and remaining water percentage (red dashed line) as functions of applied force (ADC value). Error bars represent the standard deviation across three repeated trials at each force setting. The peak efficiency was achieved at 300 ADC, identifying it as the optimal contact force for effective swabbing.

The robotic setup in Section 2.1 executed swabbing tasks using the optimized trajectory while maintaining the optimal contact force using the force-feedback control loop. Initially, the robot performed five preliminary swabbing trials using different force values represented by ADC readings ranging from 220–320 to identify the optimal swabbing pressure. The ADC value of 300 was determined to yield the best water pickup and most uniform contact across the sensor pad. Three additional swabbing trials were then conducted under this optimized configuration. The robot performed multiple swabbing trials using a force-controlled UR5e arm with real-time feedback from an embedded FSR sensor. The ideal setting was established at 300 ADC, after which the robot conducted three final trials under this optimized configuration. Figure 3 illustrates the robot’s swabbing performance under six different applied forces, represented as ADC values ranging from 220 to 320. Each force setting was tested in triplicate, and the plot displays both pickup efficiency and remaining water percentage, along with error bars reflecting standard deviation about the mean.

#### Evaluation Metrics and Statistical Analysis

Swabbing performance was assessed using three quantitative metrics: water pickup efficiency, surface coverage, and pressure consistency. These parameters were selected to capture both the effectiveness and reproducibility of sampling, which are essential for environmental monitoring in food safety applications. Water pickup efficiency (%) was calculated as Equation (3):

(3) $$\mathrm{Pickup}\;\mathrm{Efficiency}=\frac{m_{\mathrm{final}}-m_{\mathrm{initial}}}{m_{\mathrm{water}\;\mathrm{applied}}}$$

where $m_{\mathrm{final}}$ and $m_{\mathrm{initial}}$ represent sponge mass after and before swabbing, respectively, measured using a calibrated analytical balance (Sartorius, Göttingen, Germany; readability: 0.0001 g). The 1 mL applied water mass was corrected for ambient temperature density variations, verified prior to trials using a NIST-traceable reference weight. Each swabbing condition was tested in triplicate per participant/robot trial to ensure statistical robustness.

Surface coverage was quantified with a 10×10cm tactile pad segmented into discrete sensing cells. A cell was marked *activated* when contact pressure exceeded ≈0.2 N; clusters of non-activated cells were interpreted as missed zones due to insufficient force or poor technique. Coverage was computed as

$$\mathrm{Coverage}\;\left(\%\right)=100\times \frac{N_{\mathrm{activated}}}{N_{\mathrm{total}}}.$$

Raw recordings were processed in MATLAB (R2024a, MathWorks) to generate: (i) spatial heat maps of activation that visualize uniform versus incomplete contact; (ii) temporal activation profiles that track sequence and dwell; and (iii) automated flags for uneven force or systematically missed areas, enabling qualitative and quantitative assessment across replicates.

Pressure consistency was evaluated from the same pad data using two-dimensional pressure heat maps and by summarizing the distribution of applied pressure across trials. For each participant/condition, we calculated dispersion metrics (SD, variance) as indices of force uniformity, which is critical for reproducible sampling and effective residue recovery.

All analyses were conducted in R (v4.3.0) and MATLAB (R2024a). Assumptions were checked with Shapiro–Wilk (normality) and Levene’s test (homogeneity of variance). Within-subject comparisons (untrained vs. trained) used paired *t*-tests and, where repeated trials were modeled jointly, repeated-measures ANOVA. Between-group comparisons (robot vs. human means) used independent *t*-tests and one-way ANOVA as appropriate. When multiple group comparisons were performed, Tukey’s HSD controlled familywise error. Statistical significance was defined as two-sided p<0.05. Unless stated otherwise, *p* denotes a two-sided *p*-value for the specified test; for multiple comparisons, we report Tukey-adjusted values as $p_{\mathrm{adj}}$.

To complement *p*-values, effect sizes are reported alongside all tests. For *t*-tests, we report Cohen’s *d* (Hedges’ *g* with 95% CIs for pairwise Tukey contrasts). For ANOVA, we report eta-squared and omega-squared for one-way designs, and partial eta-squared for multi-factor or blocked designs shown in Equations (4) and (5) as below:

(4) $$\eta^{2}=\frac{{\mathrm{SS}}_{\mathrm{effect}}}{{\mathrm{SS}}_{\mathrm{total}}},\;\omega^{2}=\frac{{\mathrm{SS}}_{\mathrm{effect}}-df_{\mathrm{effect}}\;{\mathrm{MS}}_{\mathrm{error}}}{{\mathrm{SS}}_{\mathrm{total}}+{\mathrm{MS}}_{\mathrm{error}}},$$

(5) $$\eta_{p}^{2}=\frac{{\mathrm{SS}}_{\mathrm{effect}}}{{\mathrm{SS}}_{\mathrm{effect}}+{\mathrm{SS}}_{\mathrm{error}}}.$$

where appropriate, for unbalanced/mixed designs we also note generalized eta-squared $\eta_{G}^{2}$ [47]. Confidence intervals (95%) for $\eta^{2}$/$\eta_{p}^{2}$ were obtained via the noncentral-*F* method [48], or by bootstrap (10,000 resamples) when assumptions were marginal. Interpretive benchmarks follow [49,50].

Instrumental uncertainties from the analytical balance (±0.1 mg) and tactile pad (±0.02 N) were propagated only to quantify the instrumentation floor for single-trial derived measures (pickup efficiency, coverage); inferential tests used replicate variability, not these propagated errors. Results are reported as mean ± SD unless stated otherwise.

## 3. Results

This section compares human and robotic swabbing across three metrics: water pickup efficiency, surface coverage, and pressure consistency. We first summarize human performance (untrained vs. trained), then report the robot under the nominal force proxy (ADC≈300). Statistical tests are accompanied by effect sizes and 95% confidence intervals.

### 3.1. Water Pickup Efficiency

**Figure 4:**
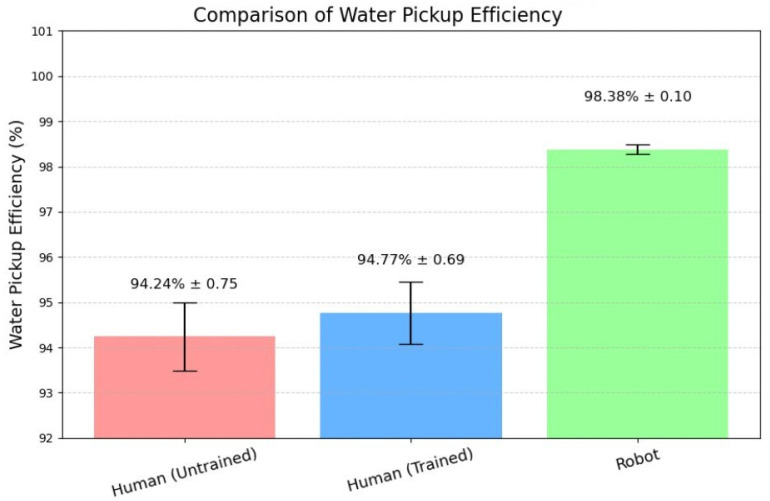
Comparison of water pickup efficiency between human participants (untrained and trained) and the robotic system. Bars represent the mean values of three replicate trials, and error bars indicate standard deviation across trials. While training improved human performance slightly, the robot outperformed both human groups with significantly higher and more consistent pickup efficiency.

Figure 4 shows the robot’s water pickup efficiency across force setpoints and the group means for human trials. The robot achieved its best performance at ADC≈300, with mean 98.4% (SD 0.1%; n=3). Human participants improved modestly with training: untrained 94.2% (SD 0.8%; n=10) to trained 94.8% (SD 0.7%; n=10). The paired training effect in our dataset was not significant (p=0.184; paired *t*-test).

An independent-samples approximation using the summary statistics yields a similar non-significant difference of (+0.60) percentage points (95% CI ([−0.11, 1.31])); Welch (t(17.69)=1.78), (p=0.091), Hedges’ (g=0.76)([−0.15, 1.68]). Comparing the robot to trained humans, the robot recovered significantly more water, as shown in Table 2: difference (+3.60) percentage points (95% CI ([3.09, 4.11])); Welch $\left(t\left(10.06\right)=15.74\right),\left(p=2.06\times 10^{-8}\right)$. The standardized effect was large with Hedges’ (g = 5.28) ([2.72, 7.83]).

### 3.2. Surface Coverage

**Figure 5:**
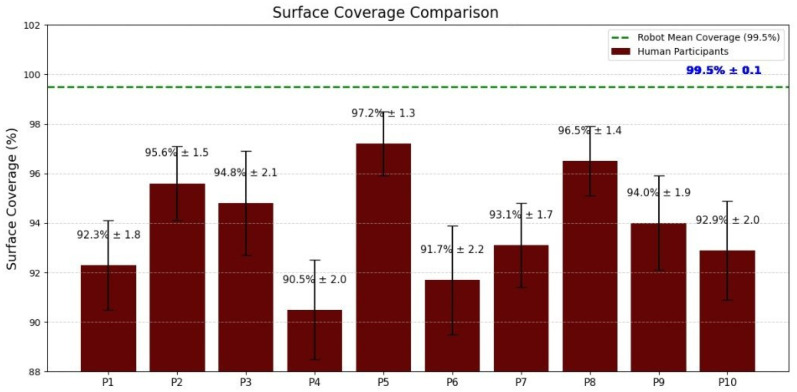
Surface coverage comparison between human participants and the robot. Each bar represents the average coverage achieved during swabbing trials. The dashed line indicates the robot’s consistently high coverage (99.5%), while human performance varied between 90–100%, reflecting natural variability and occasional missed regions.

Surface coverage was analyzed using data from the tactile sensor pad. Figure 5 presents a bar plot comparing average coverage percentages for each participant versus the robot. Trained humans achieved 90–100% surface coverage (passing the pressure threshold), often missing 1–5 sensor zones per trial. In contrast, the robot consistently achieved 99.5% average coverage with almost no missed areas. These results highlight that, even after training, humans demonstrated a natural variability in their contact patterns, while the robot ensured full surface coverage through precise motion control and feedback.

### 3.3. Pressure Consistency

**Figure 6:**
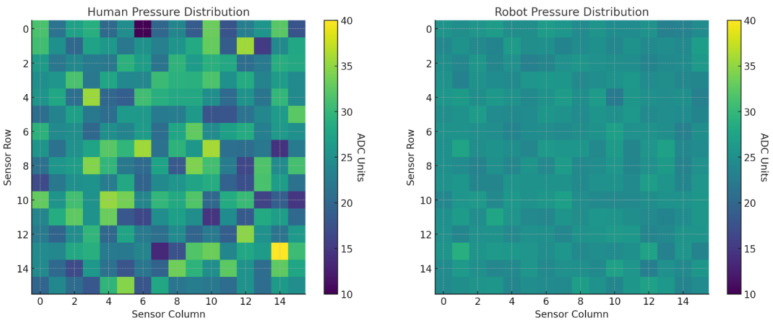
Side-by-side comparison of pressure distribution during swabbing. The left heatmap shows the variability in human-applied pressure across a 10 × 10 cm tactile sensor grid, characterized by uneven force and inconsistent contact. The right heatmap illustrates the robot’s pressure profile, which is tightly clustered around the target value (∼25 ADC units), demonstrating uniform and controlled force application.

**Figure 7:**
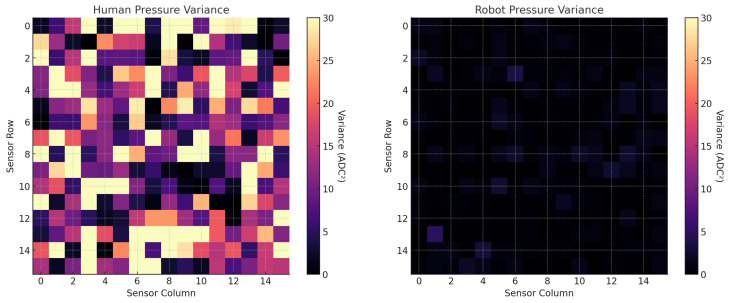
Variance maps showing inter-trial pressure inconsistency during swabbing. The left panel displays the human pressure variance across three trials, revealing high variability in contact force (up to 30 ADC) across sensor regions. The right panel shows the robot’s pressure variance, which remains minimal and uniformly distributed, confirming its consistent force application across repeated swabbing tasks. These maps highlight the robot’s superior repeatability and control compared to human operators.

Representative pressure heat maps are shown in Figure 6 and inter-trial variance maps in Figure 7. Human swabbing produced patchier, less uniform force fields, whereas the robot maintained a tightly clustered pressure profile *within the coached 270–310 ADC band*, consistent with the force–ADC calibration.

Across repeated human swabs, within-subject variability was significant (ANOVA p<0.05), with omnibus effect sizes $\eta^{2}=0.14\;\left[0.05,\;\;0.25\right]$, $\eta_{p}^{2}=0.20\;\left[0.08,\;\;0.35\right]$, and $\omega^{2}=0.12\;\left[0.04,\;\;0.22\right]$, indicating a small-to-moderate practical effect. In contrast, robot trials showed no significant inter-trial differences (ANOVA p>0.40) and small effects ($\eta^{2}=0.01\;\left[0.00,\;\;0.05\right]$, $\eta_{p}^{2}=0.02\;\left[0.00,\;\;0.08\right]$, $\omega^{2}=0.01\;\left[0.00,\;\;0.04\right]$), with inter-trial correlation exceeding r=0.98. Taken together, these results support the role of the centroid-based regulator in damping transient pivoting and maintaining target contact. In addition to *F* and *p* values, Table 3 summarizes $\eta^{2}/\omega^{2}$ and $\eta_{p}^{2}$ with 95% confidence intervals.

To consolidate the human vs. robot swabbing findings, Table 4 compares average performance between trained humans and the robot across all three-evaluation metrics. These values reflect the consistent advantage of robotic swabbing in precision, uniformity, and outcome quality.

### 3.4. Fluorescence Detection of Food Protein Soil

**Figure 8:**
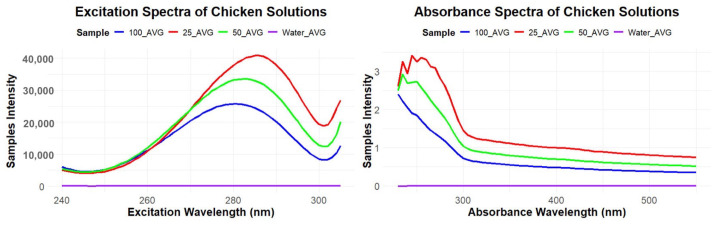
Excitation and absorbance spectra of chicken dilutions showing distinct separation; all pairs significantly different (p<0.05).

The fluorescence/absorbance detector was used to measure differences between tap water and a series of raw poultry proteins diluted with tap water. The three dilutions 1:25, 1:50, and 1:100 were analyzed to characterize detection performance. Figure 8 shows clear separation among the dilutions for both excitation and absorption spectra.

A pairwise Tukey’s HSD test showed statistically significant differences (p<0.05) between the excitation spectra (240–305 nm) of chicken dilutions and tap water. Each chicken dilution (1:25, 1:50, 1:100) exhibited higher spectral values than tap water, with the 1:25 dilution showing the greatest fluorescence intensity compared to lower concentrations (e.g., 1:100). The highest concentration (1:25) reached fluorescence peaks of approximately 42,000 counts, in contrast to the water sample peak of about 100 counts (Table 5). Tap water showed negligible absorbance, while all chicken dilutions demonstrated substantially greater absorbance. The absorbance results followed a trend similar to fluorescence: higher concentrations produced greater absorbance. Peak absorbance of chicken dilutions occurred between 230–300 nm, followed by a gradual decline across 300–550 nm (Figure 8).

To contextualize the fluorescence readout against tools already used in plants, Table 6 compares time-to-result, cost footprint, and analytical characteristics with ATP bioluminescence and protein colorimetric swabs. Briefly, ATP luminometers return results in ∼10–30 s per swab [51,52], protein swabs yield a visible color in 1–10 min [53,54], and our CCD-based fluorescence acquisition is <1 s with sample-to-answer typically <1 min [55]. Pricewise, ATP requires a handheld luminometer (order of $2.2–3.0k) plus ∼$2.7–$4.7 per-test swabs [56,57,58,59]; protein swabs are instrument-free at ∼$2.5–$5 per test [60,61]; fluorescence uses a bench spectrofluorometer (capital instrument) with UV-transparent cuvettes ∼$1–$2 per test [55,62].

Analytically, ATP reports relative light units (RLUs) from total ATP (organic residue plus cells) rather than CFU and can be influenced by lysis/sanitizer chemistry [51]; protein swabs are semi-quantitative threshold tools (e.g., ∼20–50 μg protein at 10 min) and may be affected by oxidizers [53,54]. By contrast, intrinsic fluorescence provides a *quantitative, continuous* proteinaceous signal near 280 nm (counts; Table 5). Overall, fluorescence offers a sub-minute, quantitative verification of proteinaceous residue, while ATP and protein swabs provide rapid qualitative/threshold screening; see Table 6 for the operational summary.

## 4. Limitations and Future Work

This study demonstrates the clear advantages of the force-controlled robotic swabbing system and the fluorescence-based chicken soil detection. However, several limitations should be acknowledged. First, the experiments were conducted under controlled laboratory settings using standardized surfaces and model protein soils. Although these conditions enabled precise performance comparisons between human and robotic swabbing, they may not fully represent the variability and complexities of real-world food processing environments, where surface geometries, contamination types, and environmental conditions can differ substantially. Second, the contamination sample, diluted poultry protein, does not encompass the full spectrum of potential soil types, including fatty residues, starches, biofilms, or microbial loads that may interact differently with both swabbing and fluorescence detection. Finally, the economic feasibility and cleaning requirements of deploying robotic swabbing systems in commercial plants were not evaluated and may influence industry adoption.

Future research will validate the integrated swabbing–fluorescence system under industrial production conditions, including high-moisture, high-particulate, temperature/flow variability, and diverse sanitizer residues. A fieldable implementation is underway in which the end-effector and detector are mounted on a mobile autonomous robot (AMR) with a collaborative manipulator to navigate to mapped sampling sites, estimate surface normals, and maintain the coached contact band via the centroid-based regulator. In situ calibration (force–ADC mapping and fluorescence background) and hygienic design with scripted swab changeover will be evaluated to control cross-contamination between sites.

Analytically, we will extend beyond proteinaceous residue to include microbial indicators, priority allergens, and selected chemical contaminants, with matrix-effect compensation and on-site LOD/LOQ verification on plant substrates. Results will be compared head-to-head with plant screening tools (ATP bioluminescence and protein color tests) and, where feasible, with standard microbiological assays collected in parallel.

From a systems standpoint, integration with plant automation and quality systems (e.g., digital records, dashboards, and trend analysis) will enable real-time verification and automated corrective-action triggers. Engineering work will explore modular, washable end-effectors for varied surface geometries, automated consumable handling, and adaptive trajectory planning using machine-learning controllers that adjust coverage based on tactile/fluorescence feedback.

Finally, we will quantify total cost of ownership through cost–benefit and lifecycle maintenance studies (reliability, uptime, calibration drift), and perform multi-week sanitation validation to establish long-term practicality and return on investment for large-scale food production facilities.

## 5. Discussion and Conclusions

The results of this study demonstrate a clear performance advantage of the robotic swabbing system over both trained and untrained human operators. The superior outcomes are attributable to the robot’s precise control algorithms, integrated tactile sensing, and repeatable motion execution. By maintaining optimal contact force and following a predefined coverage pattern, the robotic system consistently achieved near-complete surface engagement and higher fluid recovery rates. In contrast, human operators, even with standardized training, exhibited natural variability in applied force, swab angle, and coverage patterns, leading to occasional missed areas and reduced sampling efficiency. This variability is consistent with previous findings highlighting the limitations of manual swabbing in terms of reproducibility and microbial recovery efficiency.

From an industrial food safety perspective, consistent pressure application and complete coverage are critical factors in ensuring reliable surface sampling, as inconsistencies can lead to underestimation of microbial or residue loads and potential false assurances of hygienic conditions. By minimizing operator-dependent variability, the robotic system enhances the reliability of environmental monitoring programs, thereby strengthening sanitation verification and hazard prevention within Hazard Analysis and Critical Control Point (HACCP) frameworks.

The potential integration of fluorescence spectroscopy further broadens the system’s utility. The spectrometer provided rapid, non-contact detection of protein-based residues, effectively differentiating between contamination levels in model poultry soils. This rapid verification capability is particularly valuable for in-plant applications, where immediate feedback can facilitate corrective actions, reduce downtime, and support continuous improvement in the sanitation processes. The observed sensitivity to low protein concentrations underscores its potential as a complementary tool for validating cleaning efficacy without the need for culture-based delays. Together, the combination of force-controlled robotic swabbing and fluorescence-based residue detection presents a potential dual-modality approach to environmental monitoring. This integrated system addresses key shortcomings of manual swabbing in variability, incomplete coverage, and delayed detection, while aligning with regulatory and industry trends toward data-driven, real-time hygiene verification. Future work should investigate performance across diverse surface types, soil compositions, and real-world processing environments, as well as assess economic feasibility and integration with plant automation systems. Such advancements could further position this technology as a cornerstone of next-generation food safety monitoring strategies.

## Figures and Tables

**Table 1:** SA–KLQR reproducibility settings and selection rules (fill bracketed fields with your exact values).

Category	Parameter	Setting; Selection Rule
Timing/I/O	Servo loop $f_{\mathrm{servo}}$	[Hz]; ≥5× contact bandwidth; within UR5e real-time budget.
	Tactile/FSR rates	[Hz/Hz]; synchronous acquisition; ≈1-sample latency after denoise.
	Denoise window	[samples]; removes ADC quantization; negligible phase lag.
Koopman/EDMD	Lifted dimension dim(Ψ)	Minimal dimension that yields stable one-step prediction on calibration ramps.
	Dictionary Ψ(·)	Poly deg. 2 + $M_{\mathrm{RBF}}$ RBFs placed along path curvature.
	EDMD window/ridge λ	[s], λ= [value]; minimize one-step error; avoid overfitting.
Model bank	Regions $N_{\mathrm{reg}}$	[count]; one per path segment with distinct contact stiffness/pose coupling.
	Switching/blend β	Nearest-region with hysteresis; smooth handover using blend factor β.
LQR weights	*Q* blocks ($Q_{F},Q_{\theta },Q_{p}$)	Grid search on force RMSE under scripted ramps; reject limit cycles.
	*R* (effort)	Smallest value that suppresses chatter without slowing response.
	Integral gain $K_{I}$	Remove steady-state error without overshoot (validated on ramp).
Centroid regulator	$\tau_{D},\;\tau_{E}$	One-pass calibration: $\tau_{D}$ = 95th percentile of stable D(t); $\tau_{E}$ = onset of irregular FuzzyEn(D).

**Table 2:** Water pickup comparisons with effect sizes (summary—statistic calculations).

Comparison	Mean Diff (pp) [95% CI]	Welch *t* (df)	Hedges’ *g* [95% CI]
Robot vs. trained humans	+3.60 [3.09, 4.11]	15.74 (10.06), $p=2.06\times 10^{-8}$	5.28 [2.72, 7.83]
Trained vs. untrained ^a^	+0.60 [−0.11, 1.31]	1.78 (17.69), p=0.091	0.76 [−0.15, 1.68]

^a^ Independent-samples approximation from group means/SDs (*n* = 10 each); the paired *t*-test on the raw data was non-significant (*p* = 0.184).

**Table 3:** Omnibus ANOVA effect sizes for pressure consistency with 95% confidence intervals.

Group	$\eta^{2}$ [95% CI]	$\eta_{p}^{2}$ [95% CI]	$\omega^{2}$ [95% CI]
Human swabs (p<0.05)	0.14 [0.05, 0.25]	0.20 [0.08, 0.35]	0.12 [0.04, 0.22]
Robot trials (p>0.40)	0.01 [0.00, 0.05]	0.02 [0.00, 0.08]	0.01 [0.00, 0.04]

**Table 4:** Summary of swabbing performance comparison between the trained human and the robot arm.

Metric	Human (Trained)	Robot	Conclusion
1. Water Pickup	∼94–95%, variable across users	∼98.4%, highly consistent	Robot swabs significantly better (p<0.001)
2. Coverage	90–100%, occasional missed spots	99.5%, full pad coverage, no gaps	Robot ensures complete surface contact
3. Pressure Consistency	High variation (std/var heatmaps, ANOVA p<0.05)	Stable force near 25 (low var, ANOVA p>0.4)	Robot maintains optimal pressure uniformly

**Table 5:** Fluorescence and absorbance measurements.

Sample	Fluorescence Mean (SD) [Counts]	Absorbance Mean (SD) [AU]
Water	44(±18)	$-8.1\times 10^{-6}\;\left(\pm 9.4\times 10^{-4}\right)$
1:100 Dilution	14,000(±7400)	0.70(±0.51)
1:50 Dilution	18,000(±10,000)	1.00(±0.69)
1:25 Dilution	21,000(±13,000)	1.40(±0.83)

**Table 6:** Comparison of rapid hygiene modalities (U.S., 2025).

Modality	Time	Cost
ATP bioluminescence	10–30 s ^a^	Luminometer USD 2.2–3.0k; swab USD 2.7–4.7 ^b^
Protein swab (colorimetric)	1–10 min ^c^	No instrument; USD 2.5–5 ^d^
Intrinsic fluorescence (this work)	<1 s spectrum; <1 min workflow ^e^	Bench instrument; cuvette USD 1–2 ^f^

^a^ Manufacturer IFUs/briefs: ATP result ≤30 s (often 10–15 s) [51,52]. ^b^ Representative 2025 list/retail: luminometers [56,57]; ATP swabs [58,59]. ^c^ Protein swab IFUs and product sheets [53,54]. ^d^ Protein swab pricing ranges [60,61]. ^e^ CCD spectrum acquisition <1 s (bench spectrofluorometer) [55]. ^f^ UV-transparent disposable cuvettes (UV-grade) [62].

## Data Availability

The data presented in this study are available on request from the corresponding author.
